# Magnetic Resonance Imaging of Changes in Abdominal Compartments in Obese Diabetics during a Low-Calorie Weight-Loss Program

**DOI:** 10.1371/journal.pone.0153595

**Published:** 2016-04-25

**Authors:** Lena J. Vogt, Antje Steveling, Peter J. Meffert, Marie-Luise Kromrey, Rebecca Kessler, Norbert Hosten, Janine Krüger, Simone Gärtner, Ali A. Aghdassi, Julia Mayerle, Markus M. Lerch, Jens-Peter Kühn

**Affiliations:** 1 Department of Medicine A, University Medicine Greifswald, Greifswald, Germany; 2 Institute of Community Medicine, University Medicine Greifswald, Greifswald, Germany; 3 Department of Radiology and Neuroradiology, University Medicine Greifswald, Greifswald, Germany; National Institute of Agronomic Research, FRANCE

## Abstract

**Objectives:**

To investigate changes in the fat content of abdominal compartments and muscle area during weight loss using confounder-adjusted chemical-shift-encoded magnetic resonance imaging (MRI) in overweight diabetics.

**Methods:**

Twenty-nine obese diabetics (10/19 men/women, median age: 59.0 years, median body mass index (BMI): 34.0 kg/m^2^) prospectively joined a standardized 15-week weight-loss program (six weeks of formula diet exclusively, followed by reintroduction of regular food with gradually increasing energy content over nine weeks) over 15 weeks. All subjects underwent a standardized MRI protocol including a confounder-adjusted chemical-shift-encoded MR sequence with water/fat separation before the program as well at the end of the six weeks of formula diet and at the end of the program at 15 weeks. Fat fractions of abdominal organs and vertebral bone marrow as well as volumes of visceral and subcutaneous fat were determined. Furthermore, muscle area was evaluated using the L4/L5 method. Data were compared using the Wilcoxon signed-rank test for paired samples.

**Results:**

Median BMI decreased significantly from 34.0 kg/m^2^ to 29.9 kg/m^2^ (p < 0.001) at 15 weeks. Liver fat content was normalized (14.2% to 4.1%, p < 0.001) and vertebral bone marrow fat (57.5% to 53.6%, p = 0.018) decreased significantly throughout the program, while fat content of pancreas (9.0%), spleen (0.0%), and psoas muscle (0.0%) did not (p > 0.15). Visceral fat volume (3.2 L to 1.6 L, p < 0.001) and subcutaneous fat diameter (3.0 cm to 2.2 cm, p < 0.001) also decreased significantly. Muscle area declined by 6.8% from 243.9 cm^2^ to 226.8 cm^2^.

**Conclusion:**

MRI allows noninvasive monitoring of changes in abdominal compartments during weight loss. In overweight diabetics, weight loss leads to fat reduction in abdominal compartments, such as visceral fat, as well as liver fat and vertebral bone marrow fat while pancreas fat remains unchanged.

## Introduction

The worldwide prevalence of obesity continues to increase, and it is estimated that currently 35% of adults aged 20 years and above are overweight and 11% obese [1]. In addition to body weight, fat distribution plays an important role in assessing an individual’s risk of developing metabolic syndrome [2] because abdominal fat predisposes to insulin resistance and diabetes mellitus [3–5]. The presence of both, abdominal obesity and diabetes, may increase the risk of fat cell deposition in the liver and pancreas and compromise the metabolic function of these organs [6–11].

As a consequence, weight loss is considered an important component in the management of overweight patients with metabolic syndrome [12,13]. A decrease in visceral fat and a reduction of fat accumulation in abdominal organs may considerably improve health, as recently observed in healthy obese patients [14–18]. Some conventional programs showed promising results for healthy participants and also for participants with diabetes [13,19]. There are only a few studies that measured changes in fat content in different abdominal compartments such as visceral fat or liver fat during weight loss in obese diabetics [20–23]. Patients with insulin therapy are often excluded from these studies. Additionally, these studies were performed for periods of two to 16 weeks, and the weight-loss diets investigated ranged in energy content between 400 kcal and 1200 kcal. An energy content of less than 800 kcal is not approved for outpatient therapy. To the best of our knowledge, there is no study that monitored fat content in abdominal organs, volume of subcutaneous and visceral fat, and muscle area in each subject during a standardized weight-loss program of obese diabetics including diabetics with insulin therapy.

Chemical-shift-encoded magnetic resonance imaging (MRI) is a noninvasive technique for assessing the absolute fat content of visceral organs and for quantifying the volume of subcutaneous and visceral fat [24,25]. It is well known that fat quantification using chemical-shift-encoded MRI is confounded by several factors, such as T2* relaxation [26], T1 recovery [27,28], multispectral complexity of fat [29], eddy currents [29], and image noise [28]. Tissue fat can be quantified over the full range of 0–100% if fat/water ambiguity is resolved [30]. The fat fraction determined by chemical-shift-encoded MRI after all known confounding factors have been addressed is called proton-density fat fraction (PDFF). The PDFF is a clinically accepted imaging biomarker for tissue fat quantification [25], as demonstrated in recent studies investigation the quantification of liver and pancreatic fat. The PDFF has evolved into a robust and accurate approach for assessing tissue fat independent of the MR scanner hardware (e.g., field strength) [31,32] or software (e.g., scan parameters) [33–35] used. In addition, the PDFF calculated from chemical-shift-encoded MRI is comparable to other imaging techniques such as MR spectroscopy [33,36].

In addition, cross-sectional imaging is an accepted clinical procedure for determining muscle status [37–40]. Consequently, MRI can be used for simultaneously monitoring changes in abdominal fat content and fat volume and determining the muscle area. We hypothesized that a specific weight-loss program leads to different degrees of fat reduction in abdominal compartments and that these effects can be monitored using MRI.

Therefore, the purpose of our study was to measure the fat content of abdominal compartments and the muscle area during weight loss using confounder-adjusted chemical-shift-encoded MRI in overweight diabetics.

## Material and Methods

This prospective study was approved by the local Institutional Review Board of Greifswald University Hospital, Germany (No. BB062/12). Written informed consent was obtained before study inclusion.

### Subjects

Advertisements in several local newspapers invited subjects interested in participating in a standardized weight-loss program to contact the investigators by calling a central telephone number. Inclusion criteria were age between 18 and 70 years, known type 2 diabetes, and a body mass index (BMI) of 27 kg/m^2^ or higher. Interested subjects were excluded in case of treatment with incretin mimetic drugs, pregnancy, immobilization, severe heart, liver or renal failure, dementia, eating disorders, or alcoholism. A total of 36 subjects completed the standardized weight-loss program, but because of non-MRI-safe metal implants or pacemakers seven subjects were excluded from the MRI examination. As a result, 29 subjects, who completed the standardized weight-loss program, underwent all MRI examinations. The subjects were 19 women and ten men with a median age of 59.0 years and a median BMI of 34.0 kg/m^2^.

### Standardized weight-loss program

In the first six weeks of the standardized weight-loss program, patients received a low-calorie formula diet (OPTIFAST^®^ II Short program, Nestlé Health Science Germany). Daily consumption consisted of five sachets fully replacing normal food and corresponded to an energy content of 800 kcal. The liquid diet formula contained 96 g carbohydrates, 70 g proteins, 15 g fat, and the recommended daily amounts of vitamins and minerals. Patients were advised to drink > 2.5 liters of water and other calorie-free beverages each day. This fasting phase was followed by a four-week refeeding phase, during which regular food was reintroduced and formula diet was gradually replaced until a daily total intake of 1200 kcal was reached. During the last five weeks of the program, energy intake was gradually increased to an individual level that allowed subjects to keep their weight stable.

Once a week participants visited the study center for monitoring health status and taking part in supervised exercises. The exercise course was a combination of cardio and strength training and was part of the standardized weight-loss program (OPTIFAST^®^ II Short program, Nestlé Health Science Germany). Training intensity was increased step by step from 30% and 1–2 series with 15–25 repetitions to 70% and 1–3 series with 15–25 repetitions. The program was adjusted to individuals’ fitness levels and diseases at the discretion of the trainer. A dietitian supervised the group throughout the study and provided nutritional and behavioral counseling. To monitor the diabetes drug dosage, the participants met once a week with the study physician. The trainer, the study physician and the dietician attended a training before working with the weight-loss program to ensure standardized study implementation.

### Data collection

Blood for measuring serum transaminases and lipids was sampled in the morning after an overnight fasting period of more than eight hours. While subjects were lightly clothed and not wearing shoes, weight was measured using a digital scale (Seca 635, Hamburg, Germany) and height was measured in a standing position, not wearing shoes using a stadiometer (Seca 240, Hamburg, Germany). BMI was calculated as body weight divided by body height squared. Waist circumference was measured midway between the superior iliac spine and the lower rib margin and hip circumference at the level of the greater trochanters using a tape measure without exerting pressure on the body surface. To reduce subjective error, all measurements were taken by the same clinician. Anthropometric data were collected at baseline, after the initial six weeks of formula diet, and at the end of the 15-week weight-loss program. Blood samples were taken at baseline and at 15 weeks. MRI examinations were performed before the program as well as at 6 and 15 weeks

### Magnetic resonance imaging and data analysis

MRI was performed at 3.0 Tesla (Verio, Siemens Healthcare, Erlangen, Germany). Images were acquired in supine position using a combination of the spine coil and two body phased-array coils covering the entire abdomen.

The MRI protocol consisted of a three-echo chemical-shift-encoded sequence with water-fat separation. This sequence was acquired in the coronal plane using the following imaging parameters: TR/TE1/TE2/TE3: 6.51/1.22/2.45/4.90 ms; flip angle 9°; echo train length 1; bandwidth 914Hz/px; imaging matrix: 187x288; field of view 500x500 mm, parallel acquisition (Grappa) with an acceleration factor of 2 and 24 reference lines, slice thickness 5.0 mm, 60 slices with a distance factor of 20%. The sequence was acquired in one breathhold with a total acquisition time of 22 s.

Water and fat were separated using magnitude data (TE1 out-of-phase/ TE2 in-phase images) and information from phase images to account for water/fat ambiguities. Magnitude data were corrected for known confounders, such as T2* decay [26], T1 recovery [27,28], noise correction, and the multispectral complexity of fat [29]. The PDFF was calculated from the confounder-adjusted water- and fat images (PDFF = fat/(fat+water). PDFF calculation, including correction for known confounders [26–29], was performed offline using a home-made script for the Matlab software (version 2008; Mathworks, Natick, USA). Details of the reconstruction have been described elsewhere [41]. The accuracy of PDFF calculation using the script has been demonstrated in various methodical and clinical studies [27,33,41,42] In addition, we investigated the accuracy of PDFF determination by chemical-shift-encoded MRI in a phantom experiment. The method and results are presented in the S1 File, respectively S1 Data.

The calculated PDFF map allows quantification of the percentage organ fat content and estimation of fatty tissue volumes/volumes of tissue without fat. In addition to the PDFF map, water-only images and fat-only images were reconstructed. Image analysis was done using the Osirix software (version 4.6, Bernex, Switzerland).

The PDFF map was used to assess the fat contents of the abdominal organs, including the liver, spleen, pancreas (head, body, tail), and of the vertebral bone marrow (L1-L5). The fat fractions of the abdominal organs were measured in regions of interest (ROIs). The ROI size was adjusted to the organ but was at least 0.5 cm^2^. In regions with a very low fat content, the method sometimes yields negative fat fraction values due to image noise. Several ROIs were placed in the pancreas (head, body, tail) and in the vertebral bone marrow (L1-L5) and mean values were calculated for data analysis. Second, visceral and subcutaneous fat was determined from the fat-only images. The complete abdominal fat including subcutaneous and visceral fat from diaphragm to symphysis was segmented using a self-defined cut-off value using Osirix. The cut-off value was selected individually for each subject. From the area segmented in this way, subcutaneous fat was de-segmented manually. In addition, the amount of subcutaneous fat was assessed by measuring the supraumbilical diameter of subcutaneous fat in axial slices. Third, muscle area was assessed using a segmentation of muscle area in an axial reconstruction of a single cross-section at the level of the L4/L5 intervertebral space. Cross-sectional imaging, especially the L4/L5 approach, is an accepted technique for the evaluation of muscle area. In addition, cross-sectional imaging is widely used for assessing muscle status in epidemiologic studies [40]. Fat-only images were reconstructed in axial orientation. The muscle area was assessed in a user-defined cross-sectional image between the 4^th^ and 5^th^ lumbar vertebra.

### Statistics

Data were analyzed with STATA 13 (Stata Corp., College Station, TX, USA). The Wilcoxon signed-rank test for paired samples was used to test significant differences before and after six weeks as well as before and after the weight-loss program. All data are presented as median with 25^th^ and 75^th^ percentiles. Regression analysis was performed to evaluate associations between changes in liver fat content and age, sex, insulin therapy, and baseline liver fat. Since some relations between predictors and outcome were not linear, we used fractional polynomials to account for non-linearity [43]. To avoid overfitting, we reduced the fractional polynomials to two degrees of freedom. A p value < 0.05 was considered statistically significant.

## Results

All data patient´s are figured in S2 Data. Clinical characteristics as well as the MRI findings of the subjects at baseline and following six and 15 weeks of the dietary program are presented in Table 1. Median BMI decreased from 34.0 kg/m^2^ to 30.8 kg/m^2^ after six weeks (p < 0.001) and to 29.9 kg/m^2^ after 15 weeks (p < 0.001). Weight loss was accompanied by significant changes in anthropometric measures. Waist circumference decreased from 115 cm at baseline to 106 cm after six weeks (p < 0.001) and 102 cm after 15 weeks (p < 0.001).

**Table 1:** Anthropometric measures and measures from magnetic resonance imaging (MRI) at baseline and after six and 15 weeks.

	Baseline	After 6 weeks	After 15 weeks	Changes at 6 weeks	p	Changes at 15 weeks	p
**Anthropometric measures**							
Weight [kg]	104.3 (87.1; 116.1)	95.9 (83.3; 107.4)	90.6 (79.2; 103.7)	**-10.0 (-11.7; -8.0)**	< 0.001	**-13.2 (-18.7; -7.3)**	< 0.001
Body mass index [kg/m^2^]	34.0 (32.1; 40.8)	30.8 (28.8; 37.0)	29.9 (27.8; 34.1)	**-3.2 (-4.1; -2.8)**	< 0.001	**-4.6 (-6.2; -2.6)**	< 0.001
Waist circumference [cm]	115.0 (101.0.; 122.0)	106.0 (93.0; 117.0)	102.0 (91.0; 113.0)	**-8.0 (-11.0; -7.0)**	< 0.001	**-12.0 (-16.0; -7.0)**	< 0.001
**MRI measures**							
Liver fat content [%]	14.16 (10.36; 21.28)	5.31 (3.50; 8.38)	4.10 (2.70; 5.83)	**-8.9 (-14.1; -3.8)**	< 0.001	**-10.1 (-16.7; -4.8)**	< 0.001
Pancreas fat content [%]	9.0 (7.3; 18.4)	10.2 (7.2; 16.5)	9.0 (7.2; 13.2)	0.7 (-1.9; 2.8)	0.697	0.7 (-3.4; 1.6)	0.820
Spleen fat content [%]	0.9 (-0.4; 2.1)	1.0 (-0.4; 1.7)	0.5 (-0.5; 1.6)	-0.3 (-2.0; 1.3)	0.510	-0.6 (-1.8; 1.2)	0.524
Psoas fat content [%]	1.2 (-0.4; 2.2)	0.33 (-2.1; 1.9)	0.4 (-1.0; 1.0)	-1.3 (-3.1; 1.2)	0.073	-1.0 (-2.9; 0.8)	0.071
Vertebral bone marrow fat content [%]	57.5 (43.7; 65.1)	51.5 (42.5; 58.7)	53.6 (44.5; 61.1)	**-4.7 (-7.3; -0.3)**	< 0.001	**-4.1 (-7.6; 0.1)**	0.018
Visceral fat [cm^3^]	3175 (2176; 3629)	2156 (1623; 3121)	1608 (1350; 2119)	**-609 (-1392; -136)**	< 0.001	**-1130 (-2104; -757)**	< 0.001
Subcutaneous fat [cm]	2.97 (1.96; 3.59)	2.55 (1.72; 3.18)	2.20 (1.55; 2.96)	**-0.27 (-0.54; -0.09)**	< 0.001	**-0.49 (-0.90; -0.23)**	< 0.001
Muscle area L4/L5 [cm^2^]	243.9 (212.4; 285.1)	230.4 (210.9; 249.2)	226.8 (202.1; 258.6)	**-16.7 (-28.9; -4.5)**	< 0.001	**-16.5 (-40.0; -8.1)**	0.001

All data at baseline and after six and 15 weeks are given as medians with 25^th^ and 75^th^ percentiles in parentheses; p values refer to the Wilcoxon signed-rank test; significant changes are typeset in bold; n = 29. Negative values as observed in rare cases in psoas muscle and spleen represented errors in PDFF, caused by noise of magnitude data and fitting technique. Visceral fat volume, subcutaneous thickness and muscle area were measured in different units and therefore cannot be directly compared in their magnitude.

Most laboratory parameters changed significantly with weight loss and are summarized in Table 2. The liver enzymes serum alanine aminotransferase (ALT), serum aspartate aminotransferase (AST), and gamma-glutamyl transpeptidase (GGT) decreased significantly during the program (ALT: p < 0.001, AST: p = 0.005, GGT: p = 0.010). In addition, weight loss was associated with a decrease in triglycerides and cholesterol after 15 weeks (p = 0.001 and p = 0.003). In contrast, levels of low-density lipoprotein (LDL) and high-density lipoprotein (HDL) cholesterol did not change significantly with weight loss in our study.

**Table 2:** Laboratory data before weight loss and after 15 weeks.

Laboratory parameter	Baseline	After 15 weeks	Changes at 15 weeks	p
Triglycerides [mmol/l]	2.29 (1.58; 3.36)	1.66 (1.23; 2.34)	**-0.27 (-1.40; 0.07)**	0.001
Cholesterol [mmol/l]	5.1 (4.4; 5.5)	4.6 (4.2; 5.0)	**-0.4 (-0.8; 0.0)**	0.003
LDL cholesterol [mmol/l]	2.80 (2.38; 3.51)	2.78 (2.15; 3.36)	-0.13 (-0.48; 0.27)	0.275
HDL cholesterol [mmol/l]	1.21 (0.87; 1.48)	1.13 (1.00; 1.47)	0.02 (-0.13; 0.22)	0.449
ALT [μkatal/l]	0.58 (0.46; 0.77)	0.42 (0.36; 0.48)	**-0.16 (-0.31; -0.04)**	< 0.001
AST [μkatal/l]	0.33 (0.29; 0.43)	0.29 (0.24; 0.33)	**-0.05 (-0.12; 0.02)**	0.005
γ-GT [μkatal/l]	0.67 (0.46; 1.10)	0.43 (0.36; 0.91)	**-0.14 (-0.31; -0.05)**	0.010

All data at baseline and after 15 weeks are given as medians with 25^th^ and 75^th^ percentiles in parentheses and were calculated by Wilcoxon signed-rank test, LDL = low-density lipoprotein, HDL = high-density lipoprotein, ALT = alanine transaminases, AST = aspartate transaminase, γ-GT = gamma-glutamyl transferase; significant changes in bold, n = 29

**Fig 1:**
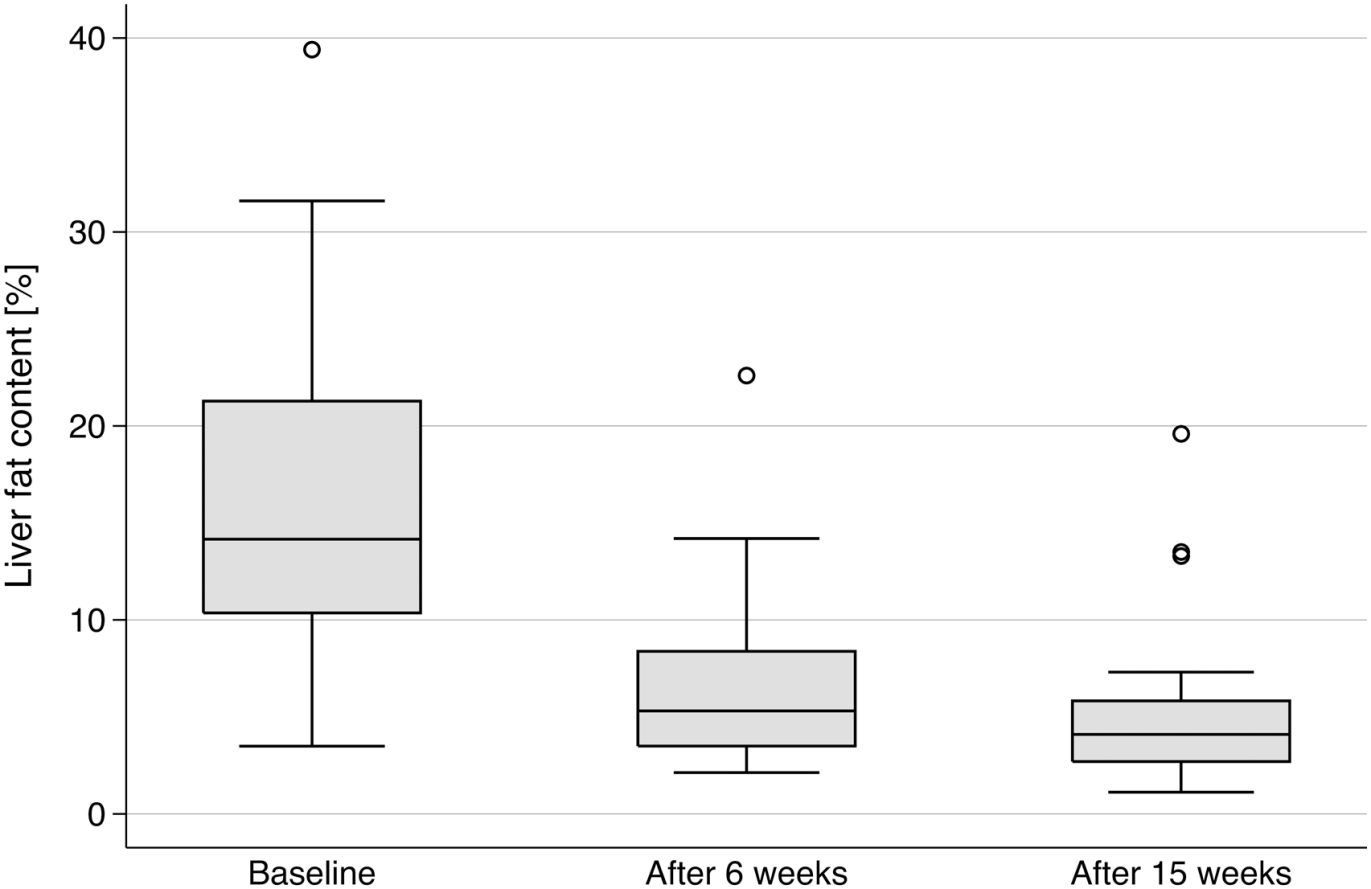
Box plots of changes in liver fat content before and after 6 weeks and 15 weeks of the weight-loss program. Most of the weight loss was observed within the first 6 weeks.

**Fig 2:**
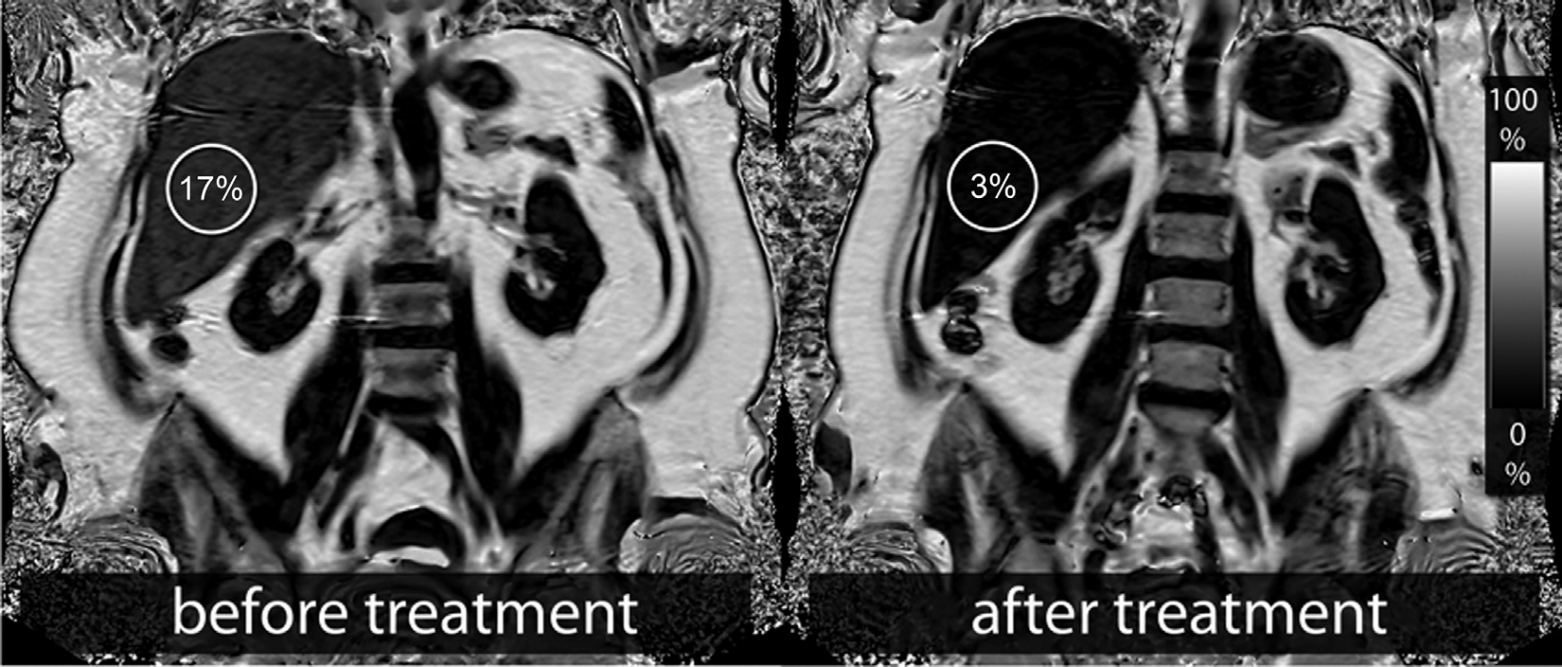
Fat fraction maps of a 54-year-old subject. This subject’s liver fat content decreased from 17% before weight loss to 3% at the end of the program, corresponding to a percentage liver-fat loss of 82%.

**Fig 3:**
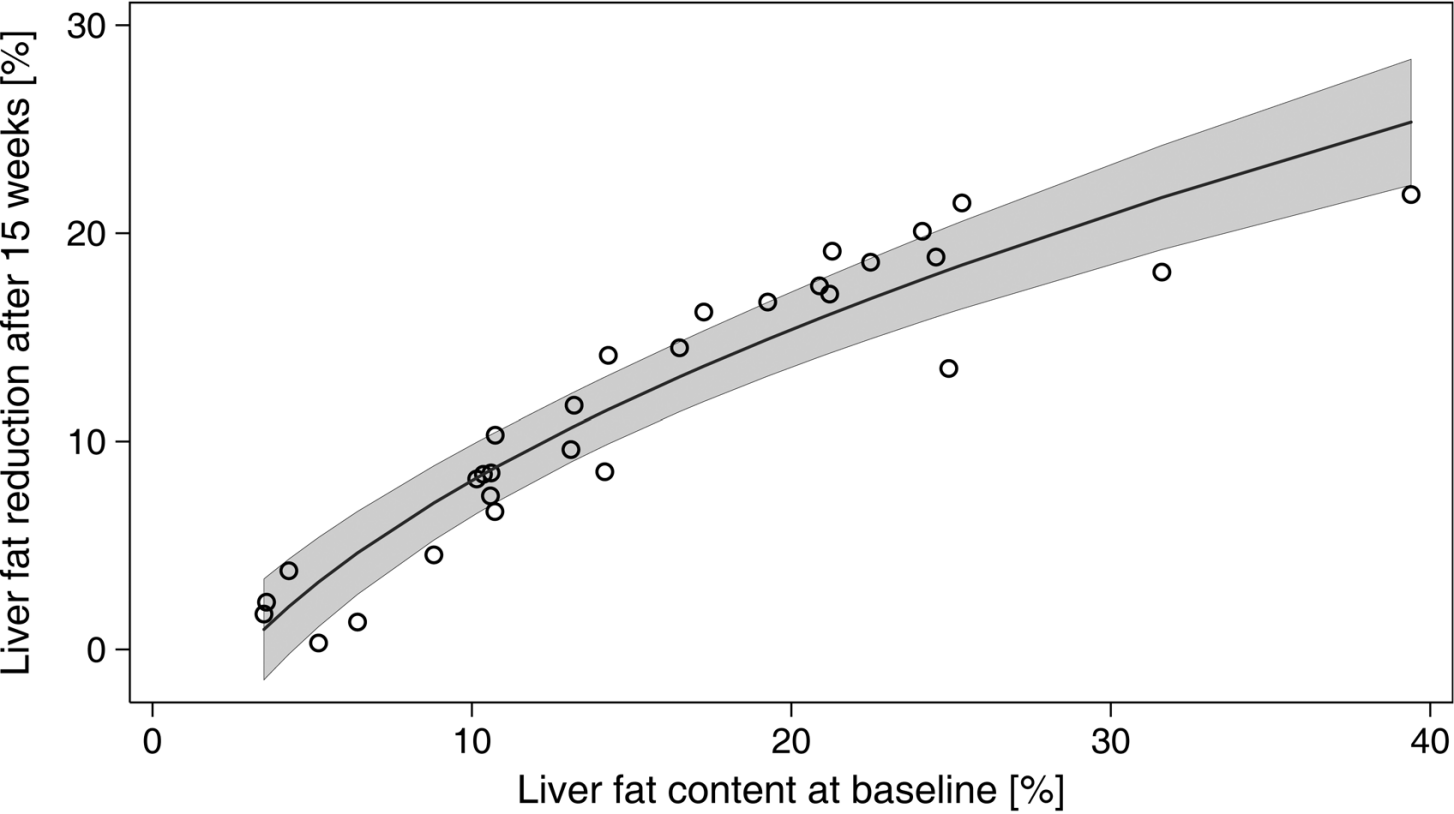
Modelled association between liver fat content at baseline and liver fat reduction at 15 weeks adjusted for age, sex, insulin therapy, and weight loss (see Table 3 for model parameters); n = 29, gray area: 95% confidence area.

Liver fat content dropped rapidly with weight loss from 14.2% at baseline to 5.3% after six weeks (p < 0.001) and was nearly normalized with 4.1% after 15 weeks (p < 0.001) (Figs 1 and 2). To investigate the influence of age, sex, insulin therapy, weight loss, and baseline value on changes in liver fat content, regression analysis was performed; the results are summarized in Table 3. Baseline liver fat was significantly (β = 18.037, p < 0.001) associated with a decrease in liver fat content (Fig 3), suggesting that subjects with a higher baseline liver fat content experienced a greater decrease in liver fat content. In our study, the factors age, sex, and insulin therapy were not significantly associated with changes in liver fat content.

**Table 3:** Regression coefficients (β) for liver fat reduction [%] after 15 weeks, R^2^ = 0.8730.

Variable	β	p	95% CI
Age [years]	-0.049	0.466	-0.187, 0.088
Female	-1.866	0.080	-3.976, 0.244
Insulin therapy	1.256	0.210	-0.759, 3.271
(Liver_0_ [%]/10)^0.5^ (polynomial)	**18.037**	**< 0.001**	15.170, 20.904
Weight change [kg]	-0.129	0.086	-0.277, 0.020

Liver_0_ = liver fat at baseline, p = significance level, CI = confidence interval, R^2^ = coefficient of determination, significant changes in bold, n = 29, note that liver_0_ has been square-root transformed because of nonlinearity of the association

While liver fat content decreased, pancreas fat content remained unchanged at a median fat content of 9.0%. A significant decline was observed in vertebral bone marrow fat content from 57.5% at baseline to 51.5% after six weeks (p < 0.001) and to 53.6% at study end (p = 0.018). Compared with the vertebral bone marrow, the fat content of spleen, and psoas muscle was very low in general and did not change significantly during the study.

**Fig 4:**
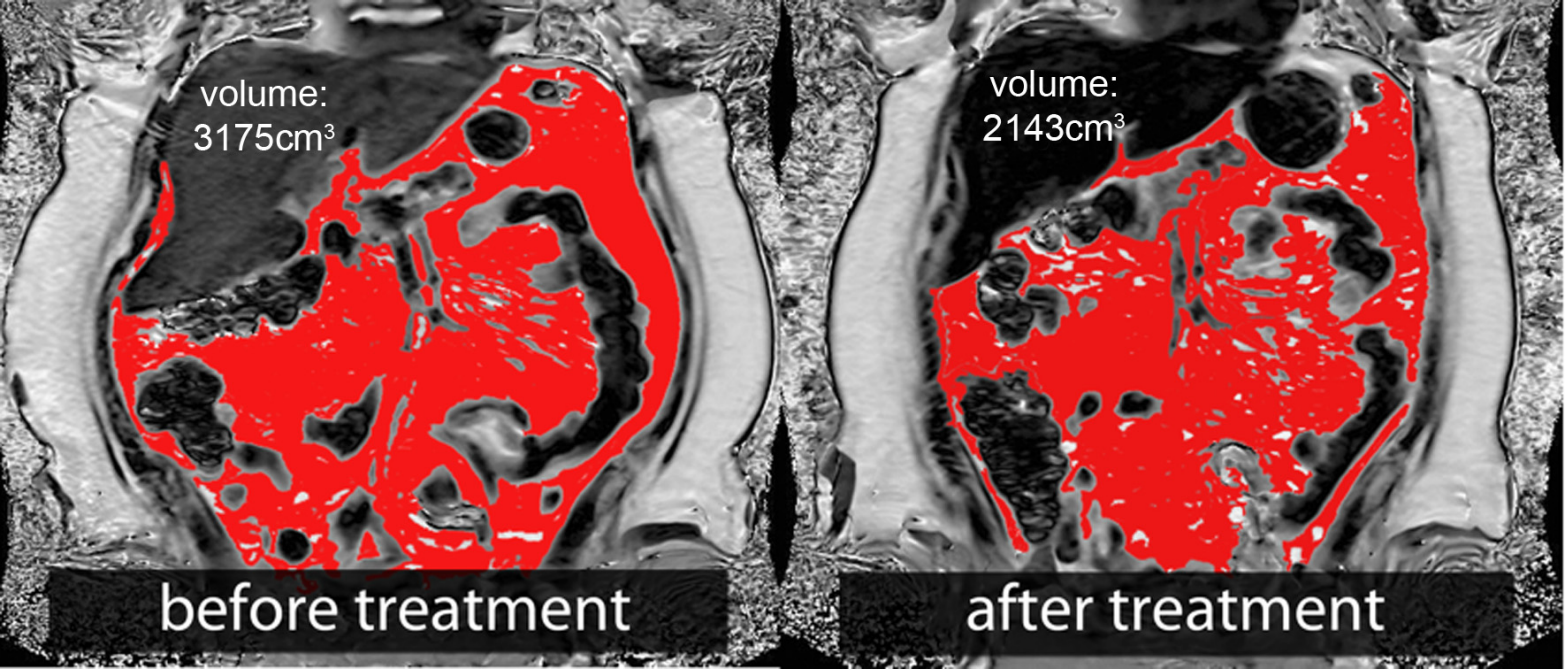
Visceral fat volume was segmented semiautomatically. Visceral fat content (same subject as Fig 2) decreased from 3175 cm^**3**^ to 2143 cm^**3**^, corresponding to a percentage visceral fat volume loss of 33%.

Visceral and subcutaneous fat volumes decreased significantly with weight loss. Median visceral fat decreased from 3175 cm^3^ at baseline to 2156 cm^3^ after six weeks (p < 0.001) and to 1608 cm^3^ after 15 weeks (p < 0.001; Fig 4). The median subcutaneous fat layer was reduced from 2.97 cm to 2.55 cm after six weeks (p < 0.001) and to 2.20 cm after 15 weeks (p < 0.001).

The median muscle area assessed using the L4/L5 approach decreased from 243.9 cm^2^ at baseline to 230.4 cm^2^ after six weeks (p < 0.001) and 226.8 cm^2^ after 15 weeks (p = 0.001).

**Fig 5:**
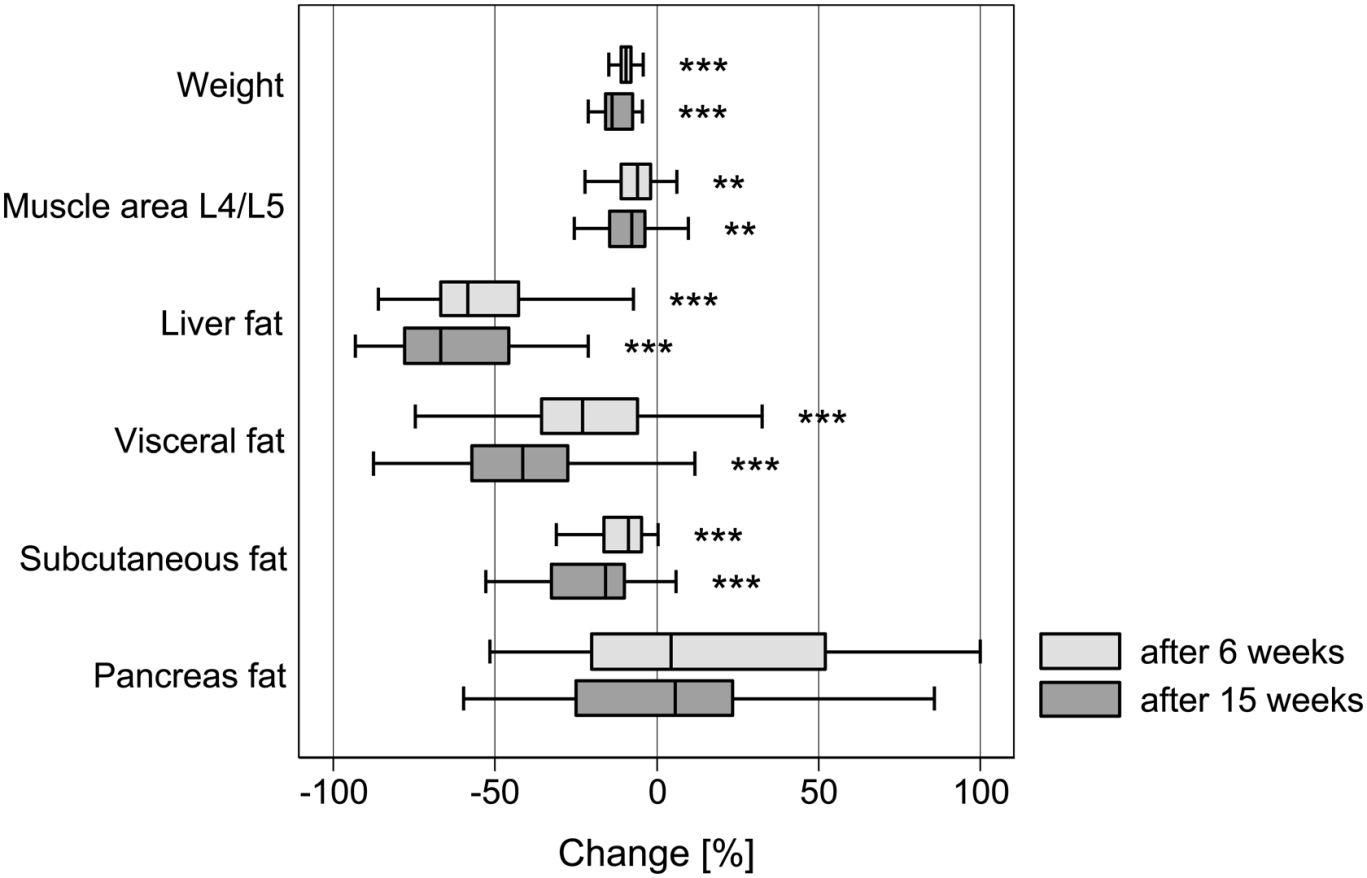
Box plots of changes in weight and abdominal compartments as determined by magnetic resonance imaging; asterisks refer to Wilcoxon signed-rank test (p < 0.01**, p < 0.001***); outliers are excluded for better presentability. Visceral fat volume, subcutaneous thickness and muscle area were measured in different units and therefore cannot be directly compared in their magnitude.

The MRI results for the individual compartments are summarized as percentage changes in Fig 5.

## Discussion

In the present study we used MRI to monitor abdominal fat compartments and evaluate changes in sarcopenia status representing muscle mass following a standardized weight-loss program in obese diabetics. Laboratory parameters such as cholesterol, triglycerides, and transaminases were significantly decreased at the end of the program. Our MRI data indicate that weight loss in obese diabetics is successful and associated with a significant reduction of liver fat and bone marrow fat content as well as visceral and subcutaneous fat, while pancreas fat content remains unchanged. As expected, the muscle area decreased significantly by 6.8% during weight reduction.

The effect of weight loss on liver fat content has been examined in obese patients with and without diabetes [14–16,18,20–23]. In these studies, percentage decreases in liver fat with different dietary programs ranged between 43.0% and 84.1%, which is consistent with our result of an approx. 67.0% reduction in obese subjects with type 2 diabetes. We observed a rapid drop in liver fat content already after the first six weeks of formula diet with 800 kcal per day. This observation is in line with the finding of Colles et al., who observed that 80% of liver fat reduction happened in the first two weeks of a diet [14]. Lifestyle changes in combination with weight loss have also been examined in patients with nonalcoholic fatty liver disease. These patients show significantly improvement in liver function tests, indicating that weight-loss programs are effective therapeutic measures in these patients [44–46]. Also in diabetics, lifestyle changes reduce the incidence of nonalcoholic fatty liver disease [45,46]. Overall, our data and recent publications indicate that a low-calorie diet and lifestyle changes rapidly reduce fatty liver disease in obese diabetics with and without insulin therapy.

In our patients, weight loss was associated with a significant decrease in vertebral bone marrow fat content of about 8.6% after six weeks and 6.1% after 15 weeks. It is well known that bone marrow contains fat and that the amount of fat depends on age [47]. Recently, a possible association between obesity and bone marrow fat content has been proposed, and investigators have hypothesized that altered bone marrow fat composition is associated with fragility fractures and diabetes [48,49]. Our results are in line with the recently published study of Schafer et al., who were the first to measure bone marrow fat before and after bariatric surgery in obese diabetic and nondiabetic women in a longitudinal design. They hypothesized that bone marrow fat increased with weight loss, because data from animals and women with anorexia nervosa showed a high content of fatty bone marrow, while total body fat content was very low [50–52]. Surprisingly, they found a decrease in bone marrow fat in diabetic patients, while it remained the same in nondiabetic patients. The authors pointed out that bone marrow may be an endocrine organ and therefore can be affected by changes in glycemic control. Because of the small sample size in our study and in the study of Schafer et al., further research is needed to confirm our results [52].

Compared with vertebral bone marrow, the lipid content of spleen, and psoas muscle was less than 1% and did not change significantly during weight reduction in our study population. Furthermore, weight loss had no influence on pancreas fat content measured by MRI. Pancreas fat content remained stable at 9.0%. Recent studies indicate that pancreas fat increases with age and BMI [53–55]. However, there is no consensus about the association between pancreatic fat and diabetes type 2. Some studies suggest that pancreas fat contributes to beta-cell dysfunction and possibly to the subsequent development of type 2 diabetes [11,16,20,53]. Other studies report no increase in pancreas fat in type 2 diabetes patients [54]. More studies are necessary to investigate the role of pancreas fat in the context of metabolic syndrome and especially its possible association with insulin resistance.

In our study, we observed a reduction of both visceral and subcutaneous fat. Visceral fat reduction was 23.1% after the first six weeks of the weight-loss program and 41.5% at the end of the 15-week Program. In contrast, subcutaneous fat reduction was 8.9% at six weeks and 15.9% at 15 weeks. Our results for visceral and subcutaneous fat reduction are in line with the study of Rossi et al., who observed a decrease in visceral fat of 31.9% and in subcutaneous fat of 13.6% in healthy obese patients after a 7% loss of total body weight. [16]. Snel et al. observed a greater decrease in subcutaneous fat in obese diabetics on insulin treatment after 16 weeks of dietary intervention [22]. This difference may be explained by the difference in energy content of the diets, which was 400 kcal in the study of Snel et al. and 800 kcal to 1200 kcal in our study. Formula diet with only 400 kcal is not approved for outpatient therapy. In contrast to the dynamics of liver fat reduction, visceral and subcutaneous fat decreased continuously over the 15 weeks in our study. This result agrees with the study of Colles et al., who observed visceral fat content over 16 weeks in healthy obese patients [14].

Liver fat content decreased more markedly during the first weeks, when subjects received a formula diet of 800 kcal per day. A disadvantage of weight loss with formula diets is that the pronounced reduction of fat volumes is associated with a moderate loss of muscle mass. However, the loss with formula diets is noticeably smaller compared with total fasting, for which a 37% reduction in muscle mass has been reported [56]. The muscle mass loss is about 4% for a standardized weight-reduction program with low-calorie diet [56]. In our study, the muscle area decreased from 241.7 cm^2^ to 225.2 cm^2^ after 15 weeks, corresponding to a loss of 6.8%. For all compartments taken together, the relative decrease in muscle area measured by MRI was about 4.9%. Hence the observed decrease in muscle area corresponds to the decrease expected on the basis of published data.

Imaging techniques allow monitoring of fat mass, fat-free mass and, muscle area [57,58]. Chemical-shift-encoded MRI is an excellent technique for assessing the percentage of tissue fat and for estimating fat volume. In a clinical setting, chemical-shift-encoded MRI is mostly used to assess liver fat content in the diagnosis of fatty liver disease. Other applications of chemical-shift-encoded MRI techniques such as quantification of osteoporosis are still under investigation [33,34,36]. In our opinion, MRI is a useful noninvasive clinical research tool for monitoring changes in fat distribution during weight loss.

One limitation of our study is that we did not investigate the long-term effects of weight loss on different body compartments. Another limitation is that we had no control group. For these reasons, further research is needed and a follow-up of our patients is planned. A third limitation is that we do not know whether our subjects complied with the dietary and exercise program when they were not present at the study center. Finally, our study is limited by the fact that we did not investigate variability of PDFF measurement by MRI as well as accuracy and precision of fat volume and muscle measurements. Recent publications indicated that this technique is independent of software and hardware for measuring fat quantity [25].

In summary, MRI is a useful clinical research tool for noninvasively monitoring changes in abdominal compartments during weight loss. Our study shows that weight loss leads to reduction of abdominal fat compartments such as visceral and subcutaneous fat as well as liver fat and vertebral bone marrow fat in overweight diabetics. In contrast, weight loss does not appear to influence pancreatic fat content and fat content of other abdominal organs in diabetics.

## Supporting Information

S1 Data Data of Phantom Measurements. (XLSX)

S2 Data Patient’s data. (XLSX)

S1 File Accuracy of Proton-Density Fat Fraction (PDFF) Determination by Chemical-Shift-Encoded MRI; phantom study. (DOCX)

## Abbreviations

MRI: magnetic resonance imaging
BMI: body mass index
LCD: low-calorie diet
ROI: region of interest
PDFF: proton-density fat fraction
