# Supplementary material for: Magnetic Resonance Imaging of Changes in Abdominal Compartments in Obese Diabetics during a Low-Calorie Weight-Loss Program
Source: PLoS One. 2016 Apr 25;11(4):e0153595. doi: 10.1371/journal.pone.0153595 (PMC4844151; doi:10.1371/journal.pone.0153595)
Supplement: S1 File — (DOCX) [file pone.0153595.s003.docx]

**S1 File. Accuracy of Proton-Density Fat Fraction (PDFF) Determination by Chemical-Shift-Encoded MRI**

Before the start of the patient study we investigated the accuracy of proton-density fat fraction (PDFF) determination by chemical-shift-encoded MRI for assessment of tissue fat content. Therefore, phantoms with different amounts of fat were constructed using veal liver and lard. Liver and lard were homogenized in a blender and placed in tanks with a volume of 125 ml. The phantoms had fat concentrations of 0, 10, 20, 30, 40, and 50%.

Chemical-shift-encoded MRI of the phantoms and reconstruction of the PDFF were performed using the same MR imager and software as for the examination of the study subjects (for details see the main text). In addition, T2-corrected multi-echo single-voxel MR spectroscopy was performed using the following scan parameters: TR: 3000ms; TE1-5: 12, 24, 36, 48, 72ms; flip angle 90°, bandwidth 1200Hz; voxel size 30x30x30mm, scan time 15 seconds.

The chemical-shift-encoded MRI sequence was acquired three times. The system was rebooted (including an additional automatic shim) between acquisitions. Figure Appendix 1 presents examples of chemical—shift-encoded MRI and MR spectroscopy of liver phantoms ranging in fat content from 0 - 50%.


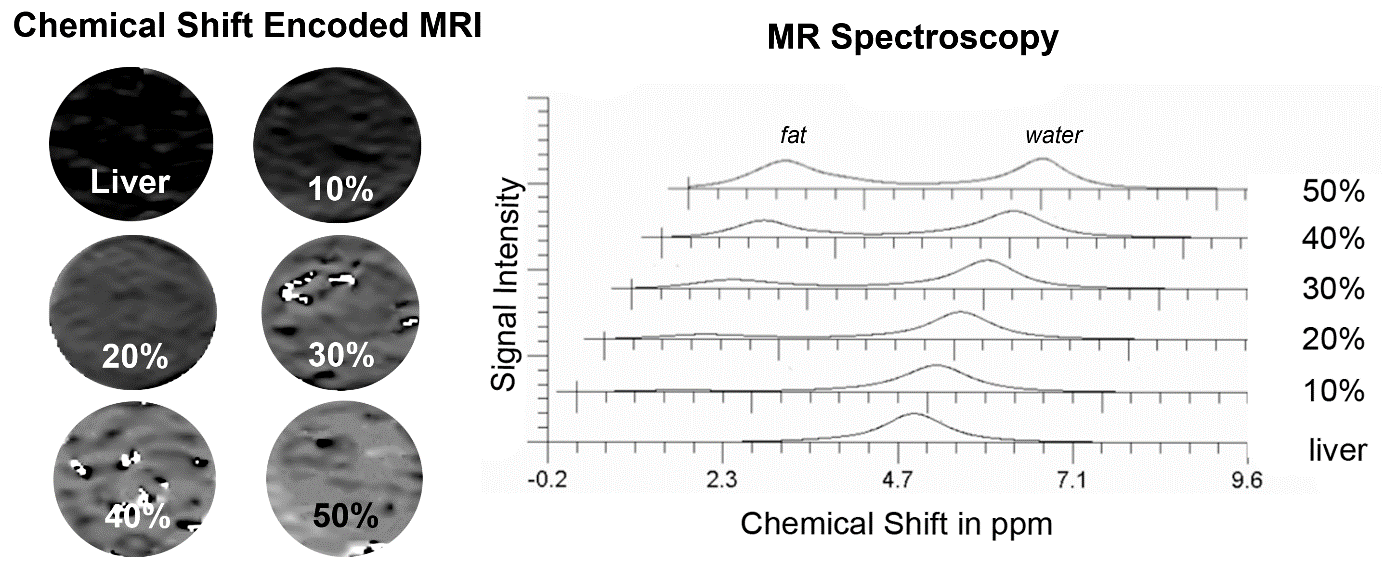


**Figure A.** Examples of PDFF determined by MRI for liver phantoms with 0 - 50% fat content (artifacts in the phantoms with 30%/40% fat correspond to small air bubbles). The right side shows the corresponding MR spectrograms for each fat content investigated.

One observer (who also analyzed images in the main study) placed regions of interest (ROIs) into the PDFF maps of each tube for each acquisition. Accuracy of PDFF determination in comparison to true phantom fat content (defined as standard of reference) was assessed using Bland-Altman analysis.

We found an absolute mean bias of 0.95% (95th CI: -0.65 – 2.55%). There was excellent correlation between PDFF determined by chemical-shift-encoded MRI and fat fraction assessed from MR spectroscopy (Spearman’s r = 0.997).

Our phantom results confirm that chemical-shift-encoded MRI is an accurate approach for assessing tissue fat with low inter-observer variability. In addition, there is excellent correlation between the fat fractions determined with different MR techniques such as chemical-shift-encoded MRI and MR spectroscopy.
